# Modeling population control via tunable sex ratio distorter gene drives in *Aedes aegypti* , an arboviral vector with homomorphic sex chromosomes

**DOI:** 10.64898/2026.07.05.736587

**Published:** 2026-08-28

**Authors:** Lauren M. Childs, Sara Shabani, Uwe Tauber, Zhijian Tu

## Abstract

*Aedes aegypti* , a major vector of arboviruses, belong to subfamily Culicinae, a diverse group of mosquitoes with homomorphic sex-determining chromosomes. Males are the heterogametic sex with a dominant male-determining locus (M locus). The M locus and its counterpart m locus are embedded in a region of suppressed recombination, with a large portion of this recombination desert showing significant molecular differentiation despite homomorphy. We developed a mathematical framework to examine M-linked genome editors that specifically target the m-chromosome during spermatogenesis, mimicking the naturally occurring sex ratio distorters (SRDs) in Culicinae that produce male-biased meiotic drives. Unlike previous models for species with heteromorphic sex chromosomes (e.g., X and Y), we incorporate features stemming from the homomorphic nature of the *Ae. aegypti* sex chromosomes such as varied linkage to the M locus, making the degree of super-Mendelian inheritance readily tunable. We evaluated *in silico* SRDs with a range of M-linkage and editing efficiencies and established the theoretical foundation for developing highly efficient SRDs that outperform several methods of population suppression. These SRDs can be tuned to mitigate impact on a neighboring population. The framework developed here is suitable for exploring SRD-mediated genetic biocontrol of numerous pest species with homomorphic sex chromosomes.

## Full Text Availability

The license terms selected by the author(s) for this preprint version do not permit archiving in PMC. The full text is available from the preprint server.

