# Supplementary material for: Modeling population control via tunable sex ratio distorter gene drives in Aedes aegypti
Source: bioRxiv. 2026 Jul 9:2026.07.05.736587. Preprint. [Version 1] doi: 10.64898/2026.07.05.736587 (PMC13370378; doi:10.64898/2026.07.05.736587)
Supplement: Supplement 1 [file media-1.pdf]

Supplementary Information for
'Modeling population control via tunable sex ratio distorter gene drives in *Aedes aegypti*'

A Supplementary Methods

Our population-level mathematical model for the dynamics of genetically distinct *Aedes* mosquito populations is represented in Figure A1 and tracks the number of juveniles J_i and male/female adults M_i, F_i of each genotype i. Each genotype is represented by two loci: one representing sex determination (M or m) and the other representing an 'editor' gene (E or a, where a is wildtype). In each generation, the juvenile population increases through birth, where the number and genotype of offspring is determined by the genotypes and abundance of the adult mating pairs (represented through the birth function $B_i(M_i, F_i)$, described in Section A.2). In a single generation, all present juveniles either develop into adults or die. The adult population increases due to the development of juveniles, which incorporates both density-dependent (via α) and density-independent (via θ) mortality as well as fitness of the various genotypes, f_i. The adult population decreases from natural mortality, μ. In contrast to juveniles, some fraction of the adult population can persist between generations, generating the potential for overlapping generations. In addition to the mortality and fitness parameters, the birth function incorporates parameters for: linkage, m-shredding efficiency, dominance coefficients, selection coefficients, and fertilized eggs per wildtype female per generation.

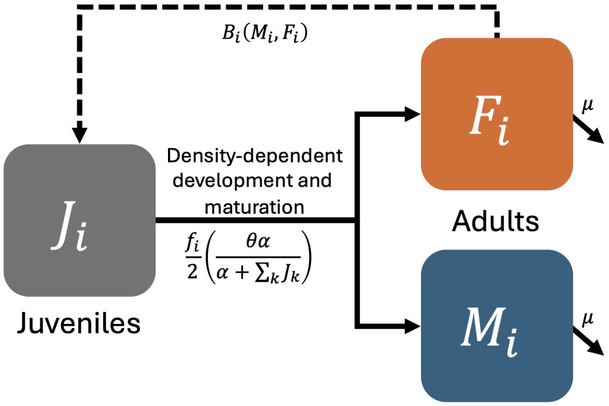

Figure A1: Schematic of conceptual model which is implemented deterministically and stochastically.

A.1 Mathematical representations

Mean-field deterministic representation. We express our compartmental model via a mean-field approximation in two ways: ordinary differential equations (ODEs) and difference equations.

The ODEs for each genotype i are given by

$$
\begin{aligned}
\overbrace{\frac{dJ_i}{dt}}^{\text{change in juvenile population}} &= \overbrace{B_i(M_i, F_i)}^{\text{birth function}} - \overbrace{J_i}^{\text{juvenile loss}} \quad , \\
\underbrace{\frac{dM_i}{dt}}_{\text{change in adult male population}} &= \underbrace{\frac{f_i}{2}\left(\frac{\theta\alpha}{\alpha + \sum_i J_i}\right) J_i}_{\text{development of surviving juveniles}} - \underbrace{\mu M_i}_{\text{adult male loss}} \quad , \\
\underbrace{\frac{dF_i}{dt}}_{\text{change in adult female population}} &= \underbrace{\frac{f_i}{2}\left(\frac{\theta\alpha}{\alpha + \sum_i J_i}\right) J_i}_{\text{development of surviving juveniles}} - \underbrace{\mu F_i}_{\text{adult female loss}} \quad .
\end{aligned} \tag{1}
$$

The difference equations are similarly given by

$$
\begin{aligned}
J_i(t+1) &= B_i(M_i(t), F_i(t)), \\
M_i(t+1) &= M_i(t) + \frac{f_i}{2}\left(\frac{\theta\alpha}{\alpha + \sum_i J_i(t)}\right) J_i(t) - \mu M_i(t), \\
F_i(t+1) &= F_i(t) + \frac{f_i}{2}\left(\frac{\theta\alpha}{\alpha + \sum_i J_i(t)}\right) J_i(t) - \mu F_i(t).
\end{aligned} \tag{2}
$$

Stochastic representation. As the deterministic representation constitutes a mean-field approximation wherein demographic fluctuations that become especially relevant for small population counts as well as temporal correlations whose importance too is amplified near extinction thresholds are neglected, we also utilize a stochastic representation of the compartmental model. Details of the stochastic implementation are found in Section A.4. Naturally, stochastic models are considerably more costly to run since obtaining decent statistics requires multiple repetitions and averaging over distinct temporal histories. Yet these repeated realizations also yield relevant probabilistic information such as estimated likelihoods of successfully eliminating undesired subpopulations.

A.2 Birth Function

The complexity of our model arises from inheritance of genetic alleles following mating and, thus, the generation of genetically diverse offspring from the birth function. As each parent produces haploid gametes, which combine to form diploid offspring upon mating, the combination of offspring from mating pairs may be more diverse than the individual parental genotypes alone. The probabilities of generating various offspring genotypes from any given parental combination are represented by a birth matrix, where two parameters determine the distribution of offspring genotype by mating pair: shredding efficiency, s, and linkage, ℓ (both discussed in detail below). The abundance of the offspring genotypes is determined by the abundance of parental genotypes in combination with the offspring probabilities from the individual mating pairs. The birth function for genotype i is given by

$$B_i = \beta \sum_{jk} M_j M_k [\text{birth matrix}],$$

where β is the average number of productive offspring per mating pair. Our standard choice of $\beta = 12$, gives a basic reproductive number of $R_m = 6$ [10]. See below for the birth matrix of all mating pairs. Furthermore, females mate only once during their lifespan, and we assume that males can mate, on average, with no more than five females. This constraint of the number of male

matings ensures the opportunity for effective population reduction, particularly when the male population is small, i.e., a single male cannot allow population persistence by mating with tens or hundreds of females in a single generation.

Birth matrix with shredding. The mating pairs (left column) produce the following distribution of offspring, which depends on the level of linkage (ℓ) and the shredding efficiency (s, which determines s_m and s_f). This matrix assumes complete shredding.

	ME-mE	ME-ma	Ma-mE	Ma-ma	mE-mE	mE-ma	ma-mE	ma-ma
ME-mE/mE-mE	s_m	0	0	0	s_f	0	0	0
ME-mE/mE-ma	$\frac{s_m}{2}$	$\frac{s_m}{2}$	0	0	$\frac{s_f}{2}$	$\frac{s_f}{2}$	0	0
ME-mE/ma-mE	$\frac{s_m}{2}$	$\frac{s_m}{2}$	0	0	$\frac{s_f}{2}$	$\frac{s_f}{2}$	0	0
ME-mE/ma-ma	0	s_m	0	0	0	s_f	0	0
ME-ma/mE-mE	$\ell\ s_m$	0	$(1-\ell)\ s_m$	0	$(1-\ell)\ s_f$	0	$\ell\ s_f$	0
ME-ma/mE-ma	$\frac{\ell\ s_m}{2}$	$\frac{\ell\ s_m}{2}$	$\frac{(1-\ell)\ s_m}{2}$	$\frac{(1-\ell)\ s_m}{2}$	$\frac{(1-\ell)\ s_f}{2}$	$\frac{(1-\ell)\ s_f}{2}$	$\frac{\ell\ s_f}{2}$	$\frac{\ell\ s_f}{2}$
ME-ma/ma-mE	$\frac{\ell\ s_m}{2}$	$\frac{\ell\ s_m}{2}$	$\frac{(1-\ell)\ s_m}{2}$	$\frac{(1-\ell)\ s_m}{2}$	$\frac{(1-\ell)\ s_f}{2}$	$\frac{(1-\ell)\ s_f}{2}$	$\frac{\ell\ s_f}{2}$	$\frac{\ell\ s_f}{2}$
ME-ma/ma-ma	0	$\ell\ s_m$	0	$(1-\ell)\ s_m$	0	$(1-\ell)\ s_f$	0	$\ell\ s_f$
Ma-mE/mE-mE	$(1-\ell)\ s_m$	0	$\ell\ s_m$	0	$\ell\ s_f$	0	$(1-\ell)\ s_f$	0
Ma-mE/mE-ma	$\frac{(1-\ell)\ s_m}{2}$	$\frac{(1-\ell)\ s_m}{2}$	$\frac{\ell\ s_m}{2}$	$\frac{\ell\ s_m}{2}$	$\frac{\ell\ s_f}{2}$	$\frac{\ell\ s_f}{2}$	$\frac{(1-\ell)\ s_f}{2}$	$\frac{(1-\ell)\ s_f}{2}$
Ma-mE/ma-mE	$\frac{(1-\ell)\ s_m}{2}$	$\frac{(1-\ell)\ s_m}{2}$	$\frac{\ell\ s_m}{2}$	$\frac{\ell\ s_m}{2}$	$\frac{\ell\ s_f}{2}$	$\frac{\ell\ s_f}{2}$	$\frac{(1-\ell)\ s_f}{2}$	$\frac{(1-\ell)\ s_f}{2}$
Ma-mE/ma-ma	0	$(1-\ell)\ s_m$	0	$\ell\ s_m$	0	$\ell\ s_f$	0	$(1-\ell)\ s_f$
Ma-ma/mE-mE	0	0	$\frac{1}{2}$	0	0	0	$\frac{1}{2}$	0
Ma-ma/mE-ma	0	0	$\frac{1}{4}$	$\frac{1}{4}$	0	0	$\frac{1}{4}$	$\frac{1}{4}$
Ma-ma/ma-mE	0	0	$\frac{1}{4}$	$\frac{1}{4}$	0	0	$\frac{1}{4}$	$\frac{1}{4}$
Ma-ma/ma-ma	0	0	0	$\frac{1}{2}$	0	0	0	$\frac{1}{2}$

Recall that in the case of shredding, where the total gamete number remains the same ($s_m + s_f = 1$), the probabilities and depend on s as follows: $s_m = \frac{1}{2-s}$, $s_f = 1 - s_m = \frac{1-s}{2-s}$.

A.3 Parameterization

Table 1: Description of key parameters and their standard values. Note that the fitness f_i is one minus the selection coefficient.

Symbol	Description	Baseline value	Range	Reference
β		12	4, 12, 24	[10]
α		200	-	[10]
θ		1	-	[10]
$1 - f_{\text{male}}$	Selection coefficient in males	0.1	[0,1]	full range possible
$1 - f_{\text{female}}$	Selection coefficient in females	0.1	[0,1]	full range possible
ℓ	Linkage	0.98 (98%)	[0.5,1]	full range possible
s	Shredding efficiency	0.90 (90%)	[0.5,1]	full range possible
μ	Natural mortality of adult mosquitoes	1	-	assumed
σ	Spillover between populations	0 (0%)	[0,0.05]	assumed
h_m	Dominance coefficient in males	1	-	[7]
h_f	Dominance coefficient in females	1	-	[7]

Linkage. A parameter of importance in the birth matrix is the linkage (ℓ) between the male-determining locus and the editor gene locus, which we allow to be variable. Linkage entails that offspring with the same combination of the sex-determining and editor gene loci as in the paternal genes are more likely, since the loci tend to be inherited together. When $\ell = 0.5$, there is neutral inheritance, as this assumes that E is autosomal and thus not linked to the sex locus. When $\ell > 0.5$, the loci are considered linked and offspring with gene combinations that are linked in those in the parental genomes are more probable. For $\ell = 1$, the linked genes are always inherited together.

Shredding. The impact on the shredding efficiency parameter (s) on the number of offspring and the extent of male bias depends on the nature of the shredding. The total number of gametes remains the same but the lost female gametes are replaced by males. The parameters s_m and s_f in the birth matrix capture the fraction of offspring generated that are either male (s_m) or female (s_f) in a single mating process. Note that when there is no shredding of female offspring ($s = 0$), then $s_m = s_f = 0.5$, and hence there is no bias towards male offspring, while for any $s > 0$, then $s_m > \frac{1}{2} > s_f$, which results in more males than females in the next generation. In the case of shredding, where the total gamete number remains the same ($s_m + s_f = 1$), the fractions (in this case, probabilities) depend on s as follows:

$$s_m = \frac{1}{2-s} \ , \qquad s_f = 1 - s_m = \frac{1-s}{2-s} \ .$$

Release fraction and type. To understand the ability of an introduced editor gene to elicit population suppression, we release male adult mosquitoes with the editor gene linked to the male-determining locus (we focus on ME-ma release as it is biologically more realistic especially when E is linked to M) and track how the population size is altered after 30 generations. We consider release fraction relative to the total adult male population at equilibrium (0.1×, 0.5×, 1×, 2×, 5×), which causes a temporary increase in the size of the adult population. We primarily consider a single release when the population is at equilibrium for the wildtype alleles, but also consider multiple releases (one per generation) of the same fraction (fixed relative to the wildtype equilibrium) up to thirty generations.

A.4 Stochastic implementation

We construct an individual-based stochastic representation extending the mean-field coupled ODE model by tracking each juvenile and adult through generation time steps subject to the following three random processes with associated prescribed probabilities:

1. **Transition out of juvenile stage:** Juveniles become adults with the density-dependent probability:
$$f_i \left(\frac{\alpha}{\alpha + \sum_i J_i} \right) .$$
All other juveniles are lost.

2. **Production of offspring via mating:** We randomly select one male and one female from the population, with the restriction that each female may participate in mating only once per generation. Based on the genotypes of the chosen male and female, there are eight different probabilities which represent the chances of producing the distinct genotypes from a particular parent pair (as determined in the columns of the birth matrix). Each offspring is generated based on a uniform random draw on these prescribed probabilities. This process is

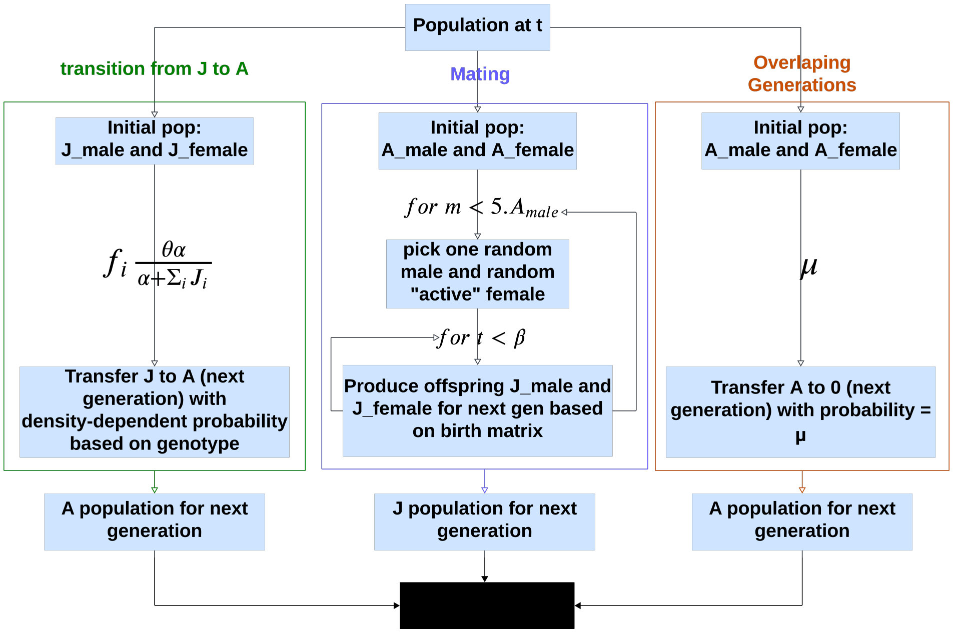

Figure A2: Flowchart of the dynamics of stochastic implementation in a single generation.

iterated β times. The resulting offspring may feature different genotypes. Next, we randomly select another male and female and repeat the this mating process five times the total adult male population number, following the assumption of the typical mating number for male individuals.

3. **Overlapping generations:** Adult individuals are removed from the population with the specified death probability, μ, which is the same for all genotypes.

These steps are detailed in the stochastic simulation flow chart found in Fig. A2. We note that we describe each mathematical generation while a biological generation (e.g., juvenile to juvenile) corresponds to two mathematical generations. This is an important detail since in the stochastic model with non-overlapping generations, genotypes released as adults will not appear in the subsequent adult mathematical generation.

A.5 Choice of parameterization for stochastic implementation

To ensure consistency between the parameterization of the deterministic and stochastic implementations of the model, we utilize the symmetric birth matrix (no bias towards male offspring or driven gene), narrow the region of our parameter space by fixing the parameters $\theta = 1$, $\alpha = 200$, and $\mu = 1$, and vary the number of produced offspring β in the range $6 \ldots 12$ in both mean-field and stochastic simulations. The default parameter values for θ, α, and μ are chosen for consistency with previous work [10]. For each set of parameters examined, for comparison to the stochastic model, we numerically solve the deterministic ODE model using Runge-Kutta and average over 200 independent Monte Carlo simulations runs for the stochastic model. Fig. A3 shows the time evolution of the total adult population over 60 generations subject to different parameters β (the number of fertilized eggs per mating per generation) for both the stochastic model simulations and the mean-field rate equation solutions. By setting the initial population to 200 individuals, equally distributed across all subpopulations, in both stochastic and mean-field simulations, we would see

that both models reach their quasi-stationary population levels after $t = 10$ generations. The ensuing quasi-stationary populations are the same in the mean-field and stochastic simulations.

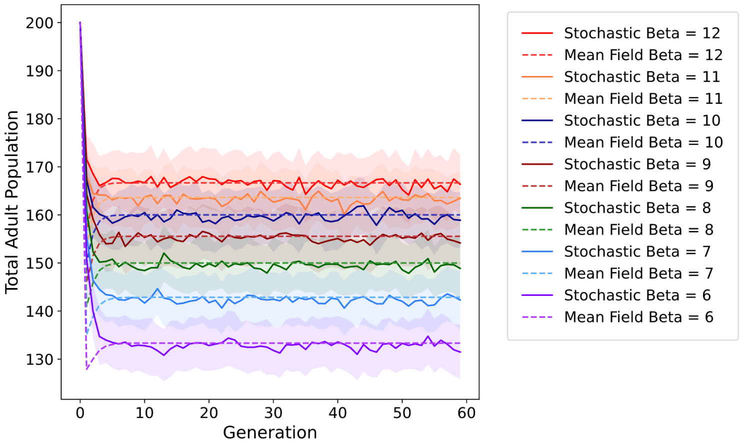

Figure A3: Adult populations in the stochastic (solid line) and mean-field (dashed) model representations across 60 generations for varying β values. Parameters: $\theta = 1$, $\mu = 1$ (non-overlapping generations), $\alpha = 200$, and symmetric birth function ($\ell = 0.5$ and $s = 0$). The initial population size is set to 200 individuals, evenly split between adult populations of each genotype: male EE, male Ea, male aE, male aa, female EE, female Ea, female aE, and female aa. Stochastic simulations are averaged over 200 independent trials.

Fig. A4 shows the (quasi-)stationary population levels after 60 generations as a function of the number of fertilized eggs per mating per generation (β). The error bars for $n = 200$ trials of the stochastic model represent the standard deviation of the stationary population data. This demonstrates that across different number of fertilized eggs the resulting stationary populations are consistent between the deterministic and stochastic models.

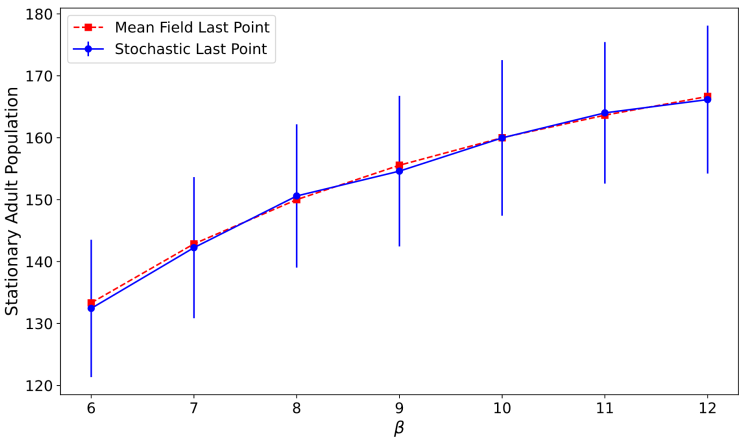

Figure A4: Total adult population after $t = 60$ generations for different β resulting from integrating the mean-field rate equations (1) and $n = 200$ independent Monte Carlo simulation runs for the corresponding stochastic model; parameters: $\theta = 1$, $\mu = 1$ $\alpha = 200$, and symmetric birth function ($\ell = 0.5$ and $s = 0$). The initial population size is set to 200 individuals, evenly split between adult populations of each genotype: male EE, male Ea, male aE, male aa, female EE, female Ea, female aE, and female aa. Stochastic results are averaged over $n = 200$ independent trials. Error bars represent the standard deviation.

B Supplemental Results

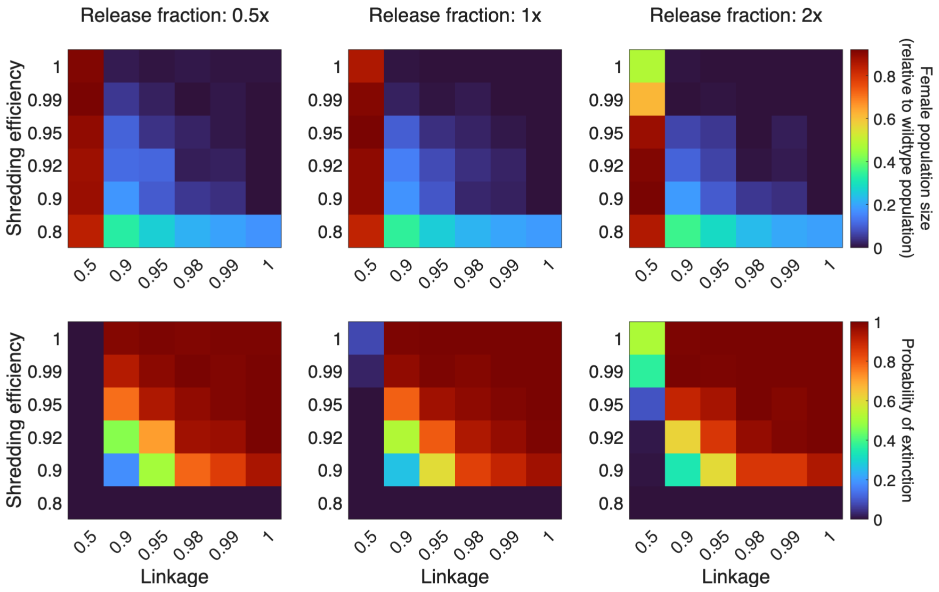

Figure A5: Female population size relative to wildtype equilibrium at 30 generations (top panels) and probability of extinction within 30 generations (bottom panels) with five releases of ME-ma males for varying linkage and shredding efficiency as obtained from our stochastic representation. From left to right, the release fraction increases (*left to right*: 0.5×, 1×, 2×). Fitness of genotypes with shredding allele is 100% that of wildtype genotype. Note the non-linear scaling of the axes. Output averaged over 200 replicate simulations with release starting after the wildtype population reached equilibrium. As it was determined that fitness costs of genotypes containing the shredding allele had minimal effect on the results for the deterministic representation for 80% to 100% fitness (Fig. A6), we assumed no fitness cost for the stochastic simulations.

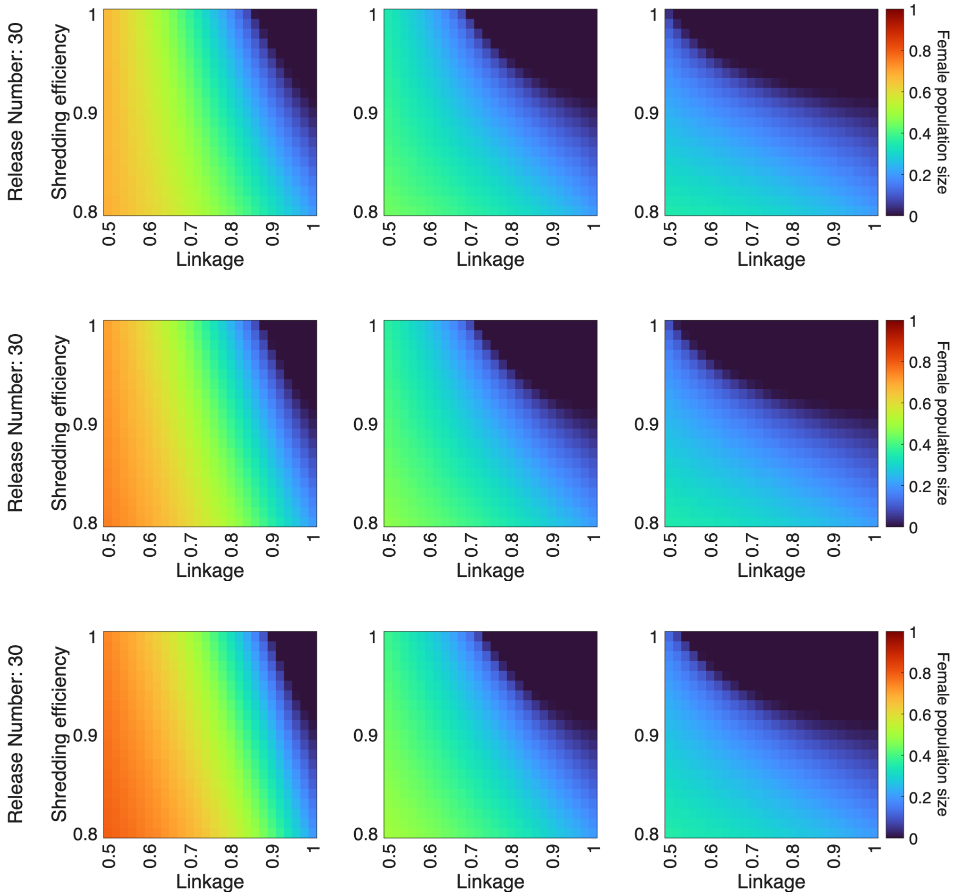

Figure A6: Female population size (relative to wildtype equilibrium) at 30 generations with 30 repeated releases of ME-ma males for varying linkage and shredding efficiency. From left to right, the release fraction increases (*left to right*: 0.1×, 0.5×, 1×). Fitness of genotypes with shredding allele: *top row:* 100%, *middle row:* 90%, *bottom row:* 80%.

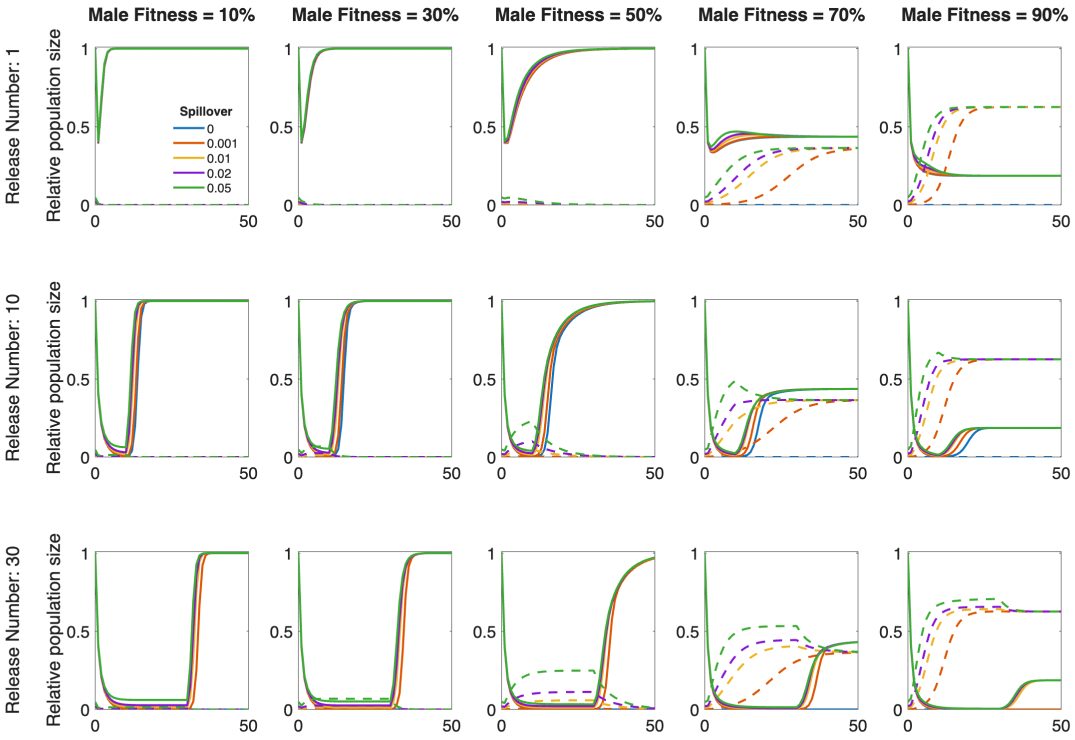

Figure A7: Dynamics of mosquito populations following a single release (top row) and repeated release (second row with 10 releases; and bottom row with 30 releases) of ME-ma males under varying linkage and spillover. Relative population size (solid lines = adult female mosquitoes relative to wildtype population at equilibrium; dashed lines = total adult male and female mosquitoes carrying at least one copy of the editor gene relative to the current total adult mosquito population) following the introduction of ME-ma males released at 2× the equilibrium male population. Fitness of male genotypes with shredding allele varies across panels from left to right: 10%, 30%, 50%, 70%, and 90%. Line color denotes spillover per generation (blue = 0%, red 0.001%, orange 0.01%, purple 0.02%, and green 0.05%). Shredding efficiency is 95%. Linkage is is 90%.

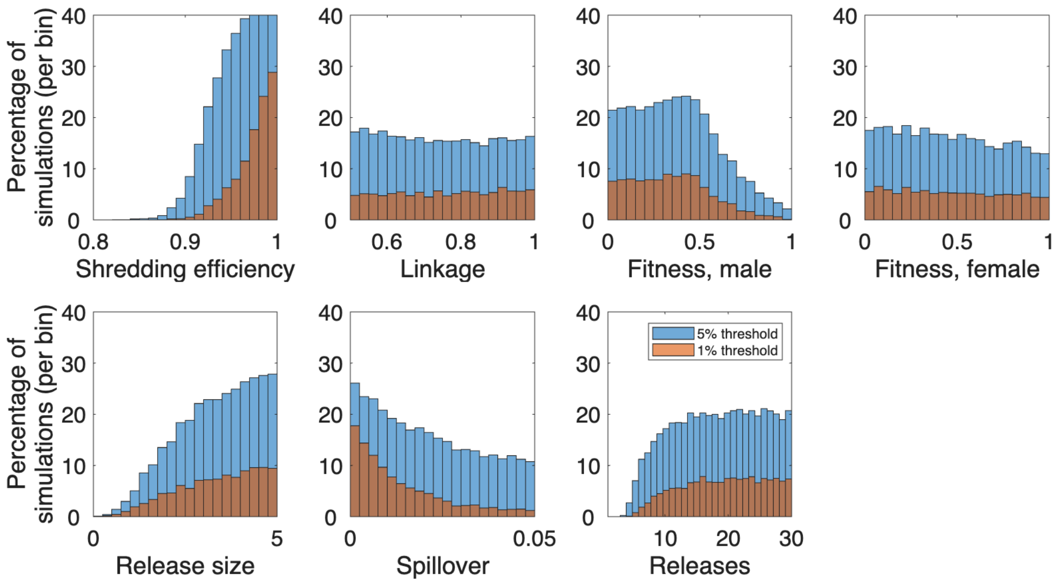

Figure A8: Values of shredding efficiency, linkage, fitness (male and female, uncorrelated), release fraction, spillover, and number of releases that lead to extinction in the population of interest (population 1) and persistence in the neighboring population (population 2) following release of ME-ma males. Blue bars show for what parameters the size of population 1 drops by 95% while the size of population 2 remains within 5% of equilibrium, and red bars show for what parameters the size of population 1 drops by 99% while the size of population 2 remains within 1% of equilibrium.

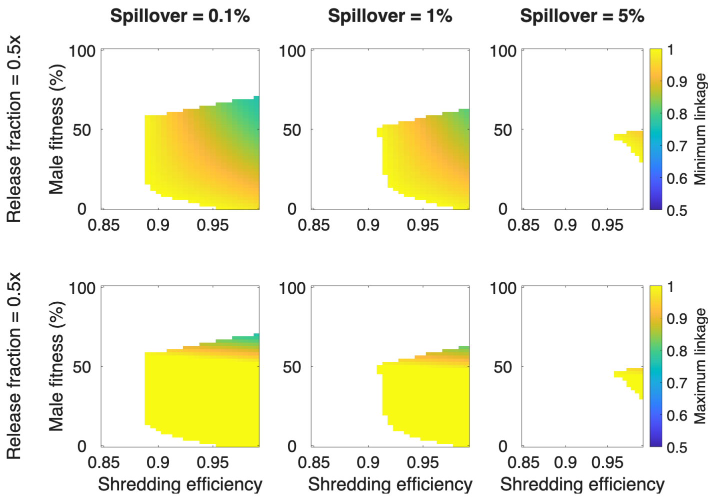

Figure A9: Minimum (top row) and maximum (bottom row) linkage for suppression of population 1 while maintaining persistence of population 2 without the presence of E alleles following 30 repeated releases of of ME-ma males with varying shredding efficiency and male fitness. Release fraction of 0.5× male wildtype equilibrium under 0.1% (left column), 1% (middle column) or 5% (right column) spillover.

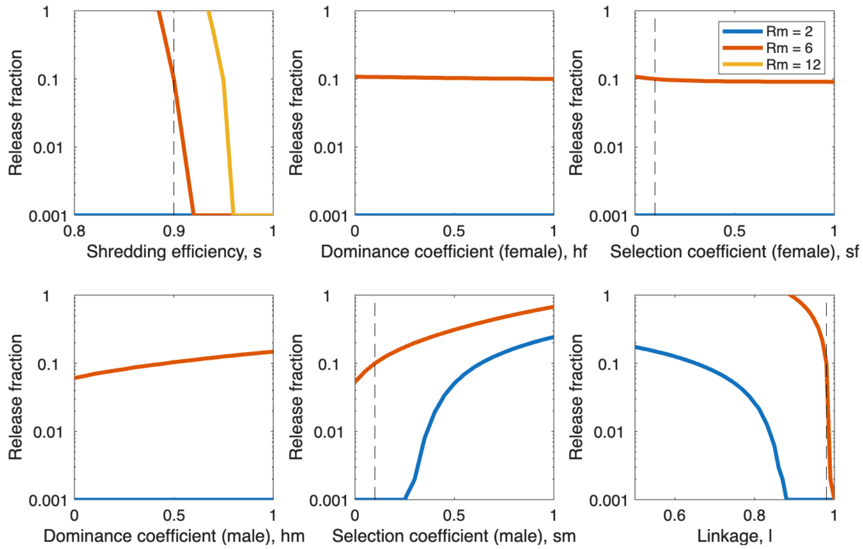

Figure A10: Univariate sensitivity analysis of key parameters. The release fraction needed to achieve 95% reduction of the population after 30 generations of repeated release. Dashed lines indicate default parameter value.
